# Quantitative imaging of gold nanoparticle distribution in a tumor-bearing mouse using benchtop x-ray fluorescence computed tomography

**DOI:** 10.1038/srep22079

**Published:** 2016-02-25

**Authors:** Nivedh Manohar, Francisco J. Reynoso, Parmeswaran Diagaradjane, Sunil Krishnan, Sang Hyun Cho

**Affiliations:** 1 Department of Radiation Physics, The University of Texas MD Anderson Cancer Center, Houston, Texas, USA; 2 Department of Radiation Oncology, The University of Texas MD Anderson Cancer Center, Houston, Texas, USA; 3 Department of Imaging Physics, The University of Texas MD Anderson Cancer Center, Houston, Texas, USA

## Abstract

X-ray fluorescence computed tomography (XFCT) is a technique that can identify, quantify, and locate elements within objects by detecting x-ray fluorescence (characteristic x-rays) stimulated by an excitation source, typically derived from a synchrotron. However, the use of a synchrotron limits practicality and accessibility of XFCT for routine biomedical imaging applications. Therefore, we have developed the ability to perform XFCT on a benchtop setting with ordinary polychromatic x-ray sources. Here, we report our postmortem study that demonstrates the use of benchtop XFCT to accurately image the distribution of gold nanoparticles (GNPs) injected into a tumor-bearing mouse. The distribution of GNPs as determined by benchtop XFCT was validated using inductively coupled plasma mass spectrometry. This investigation shows drastically enhanced sensitivity and specificity of GNP detection and quantification with benchtop XFCT, up to two orders of magnitude better than conventional x-ray CT. The results also reaffirm the unique capabilities of benchtop XFCT for simultaneous determination of the spatial distribution and concentration of nonradioactive metallic probes, such as GNPs, within the context of small animal imaging. Overall, this investigation identifies a clear path toward *in vivo* molecular imaging using benchtop XFCT techniques in conjunction with GNPs and other metallic probes.

X-ray fluorescence (XRF) analysis, which has a long history of use for biological samples^1^,^2^,^3^, operates on the premise of stimulating the production of XRF photons (i.e., characteristic x-rays) from a sample, typically using an excitation source of monochromatic x-rays, such as a synchrotron source. The resulting XRF and scattered photons are detected and analyzed in order to identify and quantify the elements contained within. Detection of XRF photons also allows a tomographic imaging technique known as x-ray fluorescence computed tomography (XFCT). With XFCT, the identity, quantity, and spatial distribution of elements within imaging objects can be simultaneously determined^4^,^5^,^6^,^7^,^8^,^9^,^10^,^11^. Owing to the spectroscopic nature of XRF-based analysis, XFCT offers fundamentally distinct advantages over attenuation/contrast-based imaging modalities (e.g., x-ray CT and its variants), such as inherent specificity due to the element-specific energies of XRF photons. Therefore, XFCT can be a powerful modality for molecular imaging of high-atomic-number (high-*Z*) probes such as metallic nanoparticles.

Among various metallic nanoparticles, gold (*Z* = 79) nanoparticles (GNPs) have attained much popularity in recent years for biomedical applications, including cancer imaging and therapy^12^,^13^,^14^,^15^,^16^,^17^,^18^. For the purpose of x-ray imaging, in particular, GNPs offer noteworthy advantages over conventional contrast agents (e.g., iodine), such as higher photoelectric interaction probability and more favorable biochemical properties (e.g., slower clearance and more specific tumor targeting). GNPs are also biologically non-reactive and chemically inert. Moreover, gold is an ideal element for XFCT of small-animal size objects (e.g., <10 cm in diameter) due to its high fluorescence yield and relatively energetic (i.e., penetrating) *K*-shell XRF photons (e.g., *K*_α1_ and *K*_α2_ at 68.8 and 67.0 keV, respectively).

In order to produce *K*-shell XRF photons from gold, the excitation x-ray energy must be above the *K*-edge of gold (80.7 keV). The implementation of XFCT with a synchrotron as a monochromatic source of x-rays above the *K*-edge of gold, while ideal, is generally regarded as impractical for routine biomedical imaging tasks, mainly because of the limited accessibility of synchrotron facilities and the high dose rate of synchrotron x-rays. We therefore explored the possibility of implementing XFCT using ordinary polychromatic x-ray sources in a benchtop setting (benchtop XFCT). While earlier investigations of benchtop XFCT produced disappointing results, our initial experimental study^19^ in which we used a pencil beam of polychromatic x-rays resulted in the first successful demonstration of the use of benchtop XFCT to image small-animal-size objects containing biologically relevant concentrations of GNPs (≤ ~1–2% by weight, or wt%). Subsequently, adopting similar pencil beam approaches, other studies^20^,^21^,^22^ explored multiplexed imaging with other metal probes and the many nuances of a pencil beam-based benchtop XFCT system. Meanwhile, we have developed a cone beam implementation of XFCT^23^,^24^ that, while technically more complex^25^, offers distinct advantages over the pencil beam approach, such as parallel XRF signal acquisition, and is also absolutely crucial for making XFCT suitable for *in vivo* imaging under the practical constraints of x-ray dose and scan time. In a more recent study, we investigated optimization of the incident polychromatic x-ray spectrum (e.g., filtration and quasi-monochromatization)^26^ as it pertains to the interplay among XRF signal production/detection, dose, and overall scanning time, in order to expedite the development of a benchtop XFCT system capable of *in vivo* imaging. The basic concepts and an overall workflow for benchtop XFCT with GNPs are illustrated in Fig. 1.

Here, we report the latest advancement in the course of benchtop XFCT development: a postmortem animal imaging study in which we have successfully applied benchtop XFCT to image a tumor-bearing mouse injected with GNPs. In contrast to previous phantom studies, this investigation was performed with an actual animal exhibiting a realistic biodistribution of GNPs in various organs/tissues and a tumor. This study clearly demonstrates the unique capabilities of benchtop XFCT under the conditions most relevant to *in vivo* imaging, charting a clear path toward *in vivo* imaging using benchtop XFCT.

## Results

All procedures of the current investigation were performed on a Swiss nude mouse bearing a human prostate cancer xenograft tumor in the right thigh.

### Cone beam computed tomography

Each of the three cone beam CT scans (pre-injection *in vivo*, post-injection *in vivo*, and post-injection postmortem) resulted in ~592 axial slices (spatial resolution of 0.15 mm × 0.15 mm and slice thickness of 0.15 mm) and took approximately 290 s to complete, resulting in an estimated x-ray dose of 39.6 cGy per CT scan. Representative CT images of the kidneys and tumor are shown in the first three panels of Fig. 2. The *in vivo* CT scan before injection of GNPs showed that the organs of interest could not be delineated using contrast. The post-injection *in vivo* CT scan showed significant contrast enhancement in the kidneys due to GNP accumulation. However, no contrast enhancement was observed elsewhere, including the tumor region. The postmortem CT scan showed even higher contrast in the kidneys, especially in the renal calyx/pelvis, but no contrast enhancement in the tumor region. As shown in Table 1, postmortem contrast-to-noise ratio (CNRs) for the left and right kidneys were 1.83 and 1.80, respectively, but the CNR for the tumor region was only 0.0295. These results highlight the limited ability of conventional x-ray CT to provide enough contrast for more biologically relevant GNP concentrations and the limited sensitivity of this technique.

### X-ray fluorescence computed tomography

For XFCT, the total imaging time for each slice (one at the level of the kidneys and the other at the level of the middle of the thigh tumor) was ~1.5 h after accounting for overhead (e.g., stage movement, stage controller initiation, and data acquisition script runtime); this resulted in an estimated x-ray dose of 74.3 cGy per slice (i.e., ~6.75 cGy per detector position, for a total of 11 detector positions per slice). After processing and reconstruction of the acquired data, each raw XFCT image, as shown in Fig. 2 (top figure, fifth column), represents 11 × 11 square pixels (121 total pixels); the spatial resolution is 2.5 mm × 2.5 mm per pixel with a slice thickness of 2.5 mm, as determined by the collimator aperture size. These XFCT images were also reproduced using bicubic approximation to create a 400 × 400 pixel representation of each slice; Fig. 2 (top figure, fourth column) displays fusion of the bicubic approximation of the raw XFCT images and the corresponding postmortem CT images from the third column. The raw data in the sinogram for each image were correlated with the average gold concentration within each pixel of the XFCT reconstruction by using the calibration curve (Fig. 3) obtained under the same imaging geometry. The kidney slice showed that the highest GNP concentration, approximately 4.35 wt%, was in the renal calyx/pelvis, and the average GNP concentration in the kidneys was 4.21 ± 0.05 wt%. The tumor slice showed that the highest GNP concentration was just 0.60 wt%, and the average GNP concentration was 0.46 ± 0.09 wt%. Also, smaller amounts of GNPs (0.25 ± 0.04 wt%) were imaged in the urethra. The results shown in Fig. 2 demonstrate that the current XFCT system clearly provides the means to visualize GNPs in the kidneys as well as in the tumor region, not only outperforming the conventional transmission CT system but also allowing the quantification of GNP concentrations in the regions of interest. CNRs calculated for the XFCT slices were compared with those for the cone beam CT slices, as shown in Table 1. The CNRs in the XFCT image for the left kidney and right kidney were 2 orders of magnitude higher than those in the CT image (174 and 189 vs. 1.83 and 1.80, respectively). Notably, the CNR for the tumor region was 15.6 in the XFCT image and 0.0295 in the CT image, further highlighting the advantage of XFCT over CT for imaging GNPs at such low concentrations.

### Inductively coupled plasma mass spectrometry analysis

The average gold content in different organs as quantified using inductively coupled plasma mass spectrometry (ICP-MS) analysis is shown in the bottom panel of Fig. 2. The gold concentration of 4.92 wt% in the kidneys was in good agreement with the XFCT results of 4.21 wt%, validating the XFCT-based quantification technique. More importantly, the much lower gold concentration in tumor tissue, as expected due to the passive targeting strategy (i.e., use of GNPs without conjugation of any tumor-targeting moiety), demonstrated close agreement between the ICP-MS (0.39 wt%) and XFCT (0.46 wt%) measurements. Note the comparison between XFCT and ICP-MS analysis was made using the data for the two most important regions of interest (i.e., the kidneys and tumor) representing relatively high and low levels of gold concentration, respectively. XFCT data for other regions of interest or organs were unavailable from the current study, mainly due to the limitations of the current benchtop XFCT setup (i.e., use of only one detector resulting in considerably lengthy overall scan time to image multiple regions of interest sequentially). Overall, these results are in general agreement with biodistribution data reported in the literature^13^ with minor differences partly attributable to the low vascularity of PC3 tumors^27^. As no perfect agreement was anticipated between the ICP-MS and XFCT results due to the differences in the analysis methods (i.e., quantification of GNPs in the whole organ vs. a single slice), this comparison was sufficient to validate the XFCT technique for at least postmortem quantification of GNPs in small animals and may serve as the basis for extending the current technique to *in vivo* imaging applications.

### CT and XFCT image fusion

For a better perspective of the imaging results, we generated a 3D volume rendering of the mouse anatomy using maximum intensity projection (OsiriX Lite; Pixmeo SARL, Geneva, Switzerland) and the postmortem CT scan data. Figure 4 shows the 3D volume-rendered postmortem image of the mouse alongside the XFCT images and representative axial CT images at the levels of the kidneys and tumor. The images demonstrate the potential for quantitative multimodal imaging of GNP distributions in an animal using XFCT and CT together.

## Discussion

In the current investigation, we have demonstrated that benchtop XFCT can be used to image a small animal injected with GNPs with higher sensitivity and specificity than conventional x-ray CT. This study was possible due to major technical advances in several aspects of benchtop XFCT, which include: (i) improved material detection limit, down to ~0.24 wt%, (ii) shorter data acquisition time compared to previous implementations of XFCT^19^,^24^, (iii) improved XRF peak extraction and attenuation correction algorithms, and (iv) absolute quantification of GNPs within each imaged pixel.

The main thrust behind these advances was the adoption of a higher-power x-ray source. After specific technical challenges resulting from the increased photon flux (reoptimizing detector parameters related to dead time and energy resolution, shielding, etc.) were successfully managed, major improvements in all aspects of the XFCT system were realized. First, the increased photon flux brought the clear advantage of faster data/signal acquisition. Second, the increased photon flux enabled the optimization of the incident x-ray spectrum via heavier filtration (i.e., with 2 mm of tin), as suggested in our previous studies^24^,^26^. The current level of filtration shifts the energy of the bulk of the 90° Compton scatter photons away from the gold *K*_α_ XRF lines, leading to improved extraction of the XRF signal. Third, the XRF peak extraction and attenuation correction algorithms were improved in two ways: fitting was improved, and statistically more stringent requirements were placed on the extraction of the net XRF signal. The added filtration with tin allowed improved fitting around the *K*_α_ XRF peaks due to the fact that the peaks were located in a more linear portion of the Compton scatter spectrum, away from any of the concavities at the base and peak of the scatter profile. This shift resulted in increased sensitivity of the peak extraction algorithm, as the fit followed the base of each peak more closely, increasing the likelihood of detecting even small increases in the net XRF signal. This improved fit also allowed statistically more stringent handling of the net XRF signal, with the requirement that the signal be at least 1.96 times the standard deviation of the background around each peak, achieving a 95% confidence level in signal extraction. In effect, the improved algorithms yielded a dramatic increase in the specificity of our benchtop XFCT system while improving the quality of the reconstructed image.

While the results of this study are encouraging, our benchtop XFCT setup needs to be further refined for routine *in vivo* imaging applications. Two of the major constraints for *in vivo* imaging are x-ray dose and overall scan time. In this investigation, a single detector had to be translated 10 times (for 11 positions) while the mouse was being rotated through 360° at each detector position, in order to obtain the necessary XRF data. This process resulted in a protracted scanning time (~1.5 h per axial slice). Although the current level of scan time/x-ray dose is deemed somewhat excessive for routine *in vivo* imaging, the current cone beam implementation of XFCT does allow parallel data acquisition with either an array of multiple detectors or a 2D pixelated detector. As a result, a drastic reduction in scan time and x-ray dose becomes immediately achievable in proportion to the number of detectors adopted. For instance, with a linear array of 11 detectors, the current scan time and x-ray dose can be reduced by more than an order of magnitude (from 90 min to ~8 min and from 74.3 cGy to ~6.75 cGy). Faster data/signal acquisition would also allow the use of smaller step sizes/projection angles, which in turn would improve the spatial resolution of XFCT images. If an improved detection limit (or sensitivity) is desired instead of a faster scan (or lower x-ray dose) under the same setup as above, the scan time/x-ray dose could be increased up to a level that is considered acceptable for *in vivo* imaging (e.g., 30 min and ~24.8 cGy). We predict that such an adjustment would result in an unprecedented detection limit for benchtop XFCT, approximately 0.05 wt% (or 500 μg gold/g) under the current conditions. The detection limit may be lowered even further (e.g., down to 0.01 wt%) by optimizing other components (e.g., detector collimation) of the system, in addition to the deployment of an array of detectors. Ideally, the cone beam benchtop XFCT setup needs to be coupled with energy-resolving 2D pixelated detectors for perfectly parallel data acquisition (or simultaneous imaging of multiple slices). However, because the energy resolution of such detectors is relatively poor compared to that of a single crystal detector as used in the current work, it is unclear whether any existing pixelated detectors can be deployed with benchtop XFCT without degrading performance. Further investigation is needed to answer this question.

In addition to the unique features of benchtop XFCT discussed above, several other important features distinguishing XFCT from other imaging modalities are important to mention. For example, it is possible to implement both micro-CT and XFCT within the same platform^28^, offering a powerful x-ray imaging tool for seamless multimodal/multiplexed imaging. Furthermore, benchtop micro-CT/XFCT, in conjunction with bioconjugated GNPs and/or other metal NP probes, may provide an unprecedented option for molecular imaging without using radiotracers. Additionally, compared to other popular molecular imaging modalities^29^, such as optical imaging and hyperpolarized magnetic resonance imaging, benchtop XFCT retains all the advantages of using x-rays and NP probes, such as superior depth penetration (several cm vs. a few mm) and longer biological half-lives (hours to days vs. seconds to minutes); these characteristics facilitate tomographic imaging of larger objects (e.g., up to ~10 cm in diameter) and longitudinal molecular imaging studies, respectively.

Noting that *in vivo* implementation of benchtop XFCT would be subject to constraints on both dose to the animal and time that the animal can remain anesthetized, the primary focus will be on deployment of additional detectors or an array of detectors to make XFCT more efficient. Also, simultaneous CT/XFCT scan protocols will be investigated to make better use of x-ray dose; for instance, an optimized *in vivo* imaging study may not require three CT scans as performed in this postmortem study. Moreover, active targeting strategies, such as conjugation of GNPs to tumor-targeting moieties^30^, may be explored to increase the GNP uptake/accumulation in the tumor to potentially allow more detailed target-specific imaging of intratumoral distributions using XFCT.

In summary, after several iterations of development and optimization, benchtop XFCT is nearing the stage of *in vivo* translation for small-animal imaging. This postmortem mouse study has highlighted the unique capabilities of benchtop XFCT and, despite a number of remaining technical hurdles, has clearly demonstrated the feasibility of quantitative imaging of small animals injected with GNPs using a benchtop cone beam XFCT system. This investigation has also identified a clear path toward the implementation of benchtop XFCT for routine preclinical *in vivo* imaging.

## Methods

### Animal and tumor

The animal used in this study was a male Swiss nude mouse bearing a tumor (human prostate cancer xenograft) that had been induced via subcutaneous injection of 0.05 mL of 2–3 × 10^6^ PC3 cells (PC3 cell line; American Type Culture Collection, Manassas, VA) in phosphate-buffered saline (PBS) into the right thigh. At the time of this investigation, the mouse was 8 weeks old, weighed 30 g, and the tumor had reached approximately 8 mm in diameter. All experimental methods regarding the animal were carried out in accordance with protocols and guidelines approved by the Institutional Animal Care and Use Committee of The University of Texas MD Anderson Cancer Center.

### Gold nanoparticle injection

A GNP-containing solution for injection was prepared using dried, biocompatible, 1.9-nm-diameter GNPs (AuroVist; Nanoprobes Inc., Yaphank, NY) according to manufacturer-provided instructions. First, the GNPs provided in one vial (containing 40 mg of gold) were dissolved in 0.19 mL of PBS. The solution was then filtered through a 0.22-μm centrifugal filter and centrifuged at 15,000 *g* for 8 min. A second filtration was performed with 0.01 mL of PBS. The final product contained gold at a concentration of 200 mg/mL (40 mg of gold suspended in 0.20 mL of PBS). The GNPs were not conjugated to any tumor-targeting moieties in order to produce a biodistribution that would allow comparison with results published in the literature^13^ in which such a passive targeting strategy was executed; therefore, uptake/accumulation of GNPs in the tumor was expected to be low as it relies mainly upon leaky tumor vasculature and the associated enhanced permeability and retention (EPR) effect.

### Cone beam computed tomography

A small-animal irradiator (X-RAD 225Cx; Precision X-Ray Inc., North Branford, CT), featuring a source-to-isocenter distance of 30 cm, source-to-detector distance of 60 cm, and a field-of-view of 10 cm × 10 cm, was used for cone beam CT of the mouse. A holder was devised to allow the mouse to be restrained in a prone position and to be supplied with anesthetic gas (isoflurane, 0.5 to 2%) via inhalation during scanning. Scan parameters were set to 50-kVp accelerating potential at 3 mA of beam current and filtration by 2 mm of aluminum. Under these conditions, the dose rate at the isocenter was estimated^31^ to be 8.20 cGy/min. Manufacturer-provided software was utilized for both image acquisition and reconstruction. No deliberate attempt was made to reduce the dose rate for the current cone beam CT study in order to ensure the best image quality achievable under the given setting.

### X-ray fluorescence computed tomography

**Figure 1:**
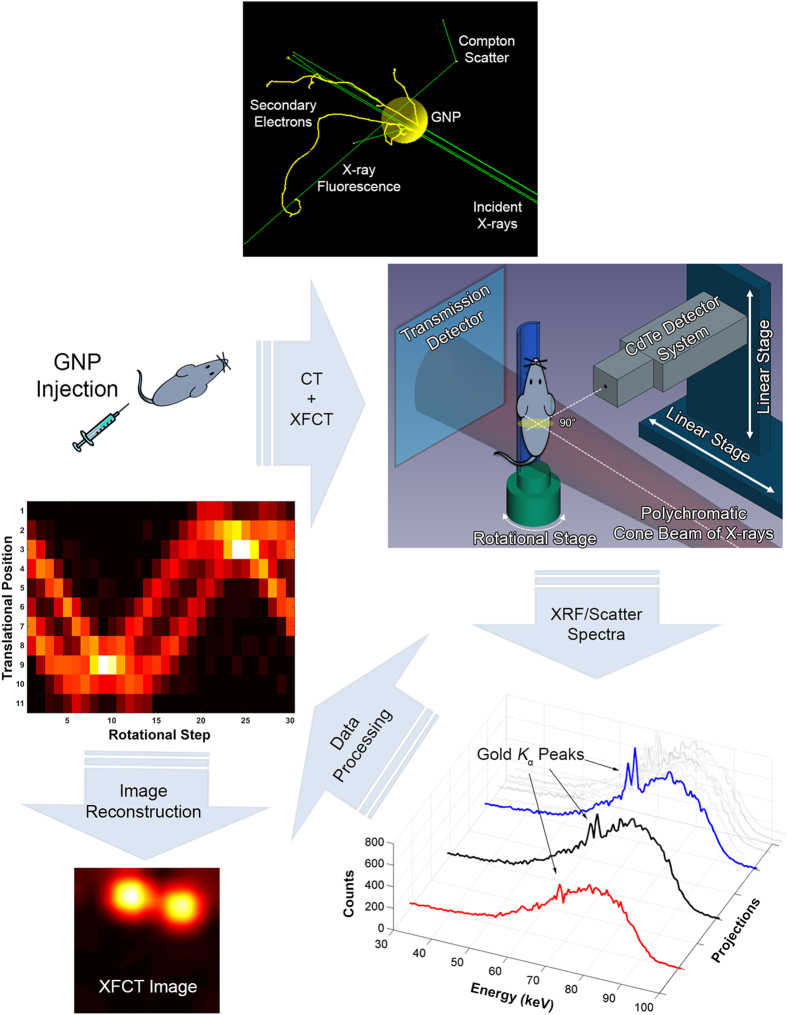
Schematic representation of benchtop XFCT imaging of a mouse injected with GNPs. As illustrated in the top image (obtained using the Geant4 toolkit)^34^, photon irradiation of GNPs results in the production of secondary electrons, scattered photons, and XRF photons (e.g., gold *K*_α1_ and *K*_α2_ at 68.8 and 67.0 keV, respectively) when the energy of photons exceeds the photoelectric absorption edges of gold (e.g., *K*-edge of 80.7 keV). Thus, gold XRF photons can be produced and detected for XFCT imaging, when a mouse injected with GNPs is irradiated by a diagnostic energy x-ray beam (e.g., 125 kVp). In the current work, the vertically oriented mouse was irradiated by a polychromatic cone beam of 125 kVp x-rays filtered by 2 mm of tin. An energy-resolving cadmium-telluride (CdTe) x-ray detector was placed at 90° with respect to the beam direction to collect the XRF/scatter spectrum at each rotational angle and translational position of the mouse. The acquired data were processed to reconstruct axial XFCT images. Note, while not used for the current work, a transmission detector (as depicted in the figure) can be added to the benchtop XFCT setup for simultaneous micro-CT/XFCT in the same platform.

**Figure 2:**
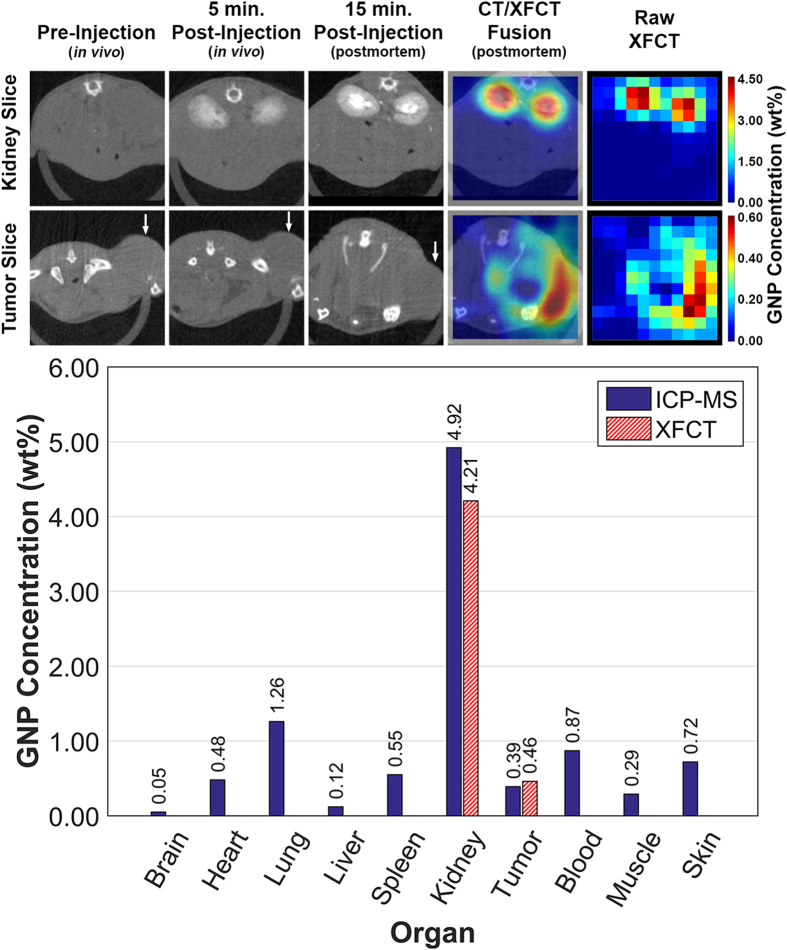
Reconstructed axial CT slices at various time-points and XFCT images at the levels of the kidneys and tumor (white arrow); gold concentrations in various organs quantified using ICP-MS analysis and XFCT. In the top panel, the first, second, and third columns demonstrate that GNP accumulation over time can be visualized using CT in the kidneys, but no such accumulation over time is visible in the tumor. Note that the tumor geometry in the postmortem CT scan differs from the tumor geometry in the other two scans due to different mouse positioning on the holder. The fourth column in the top panel shows interpolated XFCT images fused with corresponding CT images, while the fifth column shows the raw XFCT images only. The tumor XFCT images have been scaled such that the brightest pixel corresponds to a GNP concentration of 0.60 wt%, while the brightest pixel in the kidney XFCT images corresponds to a concentration of 4.35 wt%. The average GNP concentrations imaged by XFCT in the tumor and the kidneys (the only two regions of interest that were imaged by XFCT due to the limitations of the current setup), were ~0.46 wt% and ~4.21 wt%, respectively. The bar graph in the bottom panel displays these, as well as the gold concentrations in different organs quantified using ICP-MS analysis; in particular, the average gold concentration in the tumor and kidneys were 0.39 wt% and 4.92 wt%, respectively, thereby validating the XFCT results.

A general illustration of the XFCT setup, with the exception of a transmission detector, is depicted in Fig. 1. A clinical orthovoltage x-ray unit (RT-250; Philips Healthcare, East Haven, CT) was used as the excitation source for XFCT. This machine was operated at an accelerating potential of 125 kVp and a beam current of 25 mA. The resulting polychromatic x-rays were filtered using 2 mm of tin to produce a favorable incident spectrum that would allow high sensitivity (i.e., low detection limit) while still maintaining a reasonable total scan time under the given conditions^26^. The dose rate at the isocenter, which was at a distance of 50 cm from the source, was estimated^31^ to be 0.826 cGy/min. The filtered beam was collimated using a 3-cm-diameter applicator in order to cover the chosen region of interest on the mouse. A compact, thermoelectrically cooled cadmium telluride (CdTe) gamma and x-ray detector (XR-100 T-CdTe; Amptek Inc., Bedford, MA) with an energy resolution of approximately 700 eV (full width at half maximum) in the energy range of interest (~68 keV) was utilized. The detector was placed at 90° with respect to the beam direction, behind a 2.5-mm-diameter, 4-cm-thick pinhole collimator made of lead. A combination of detector assembly horizontal translation (3-mm steps) and mouse rotation (12° steps) was used to obtain data required to reconstruct each axial slice of interest. Specifically, the mouse was secured in a vertical orientation, and rotated through a full 360° at each of the 11 translational positions of the detector assembly. Therefore, there were 30 projections per translational position, for a total of 330 projections at which XRF/scatter spectra were acquired by the CdTe detector.

### Experimental procedure and imaging

After the mouse was anesthetized and before injection of the prepared GNP-containing solution, a cone beam CT scan of the mouse was performed according to the procedure described above. All 0.20 mL of the GNP-containing solution was then injected into the mouse with a 25-gauge needle via the tail vein, for an injected dose of 1.33 mg of Au/g of body weight. Another cone beam CT scan of the mouse was done (*t* = 5 min after injection) to attempt to visualize the *in vivo* distribution of GNPs by means of contrast enhancement^17^. The mouse was then euthanized via cervical dislocation (*t* = 15 min), and a third CT scan was performed postmortem. The axial slice of interest at the level of the kidneys was located using anatomical features in the postmortem CT images. The surface of the mouse was then marked at this location and at the location of the thigh tumor, in preparation for XFCT. The mouse was transported from the small animal irradiator to the room containing the orthovoltage x-ray unit around which the XFCT system was configured and XFCT scans of the two axial slices of interest were performed according to the procedure described in the previous section. The kidney slice was imaged first (started at approximately *t* = 45 min), followed by the tumor slice (started at approximately *t* = 140 min).

### Data processing and XFCT image reconstruction

After the two XFCT scans were completed, the data were processed separately for each slice. First, the individual XRF/scatter spectra obtained at each projection were corrected for the energy-dependent detection efficiency of the detector^32^. Subsequently, for each spectrum, we applied an empirical 6^th^-order polynomial fit to the Compton scatter background while excluding the energy bins where the gold *K*_α_ XRF peaks (68.8 and 67.0 keV) would occur; the high order of the polynomials minimized susceptibility to yielding atypical fits, especially when the data were noisy (i.e., the counts were low), and allowed robust handling of the variety of scatter profiles that were observed. This procedure is highlighted in Fig. 5. Using this fit, we extracted the net XRF signal from each spectrum while only considering signal values that were at least 1.96 times the standard deviation of the background around each peak, in order to ensure that the net signal extracted was within the 95% confidence interval. In order to correct for attenuation within the animal, the ratio of the net XRF signal to the Compton scatter counts was used as an approximate internal measure of attenuation^33^, requiring no *a priori* information regarding the attenuation properties of the object (i.e., the mouse). This approach allows absolute quantification of GNPs within the animal, as Compton scatter serves as an internal probe of attenuation. XFCT images were then reconstructed by applying a filtered back projection tomographic reconstruction algorithm to the corrected XRF signals for each projection.

The CNRs for analysis of the CT data were calculated as 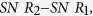 where 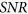 represents the signal-to-noise ratio for each region of interest. For the CT data, *SNR*_1_ and *SNR*_2_ are the signal-to-noise ratios for each region of interest before and after injection of GNPs, respectively. In the case of XFCT images, which were only obtained after injection of GNPs, the CNR was defined simply as the signal-to-noise ratio of each region of interest; this is because the signal-to-noise ratio is zero for regions which do not exhibit XRF signal.

### XFCT calibration

**Figure 3:**
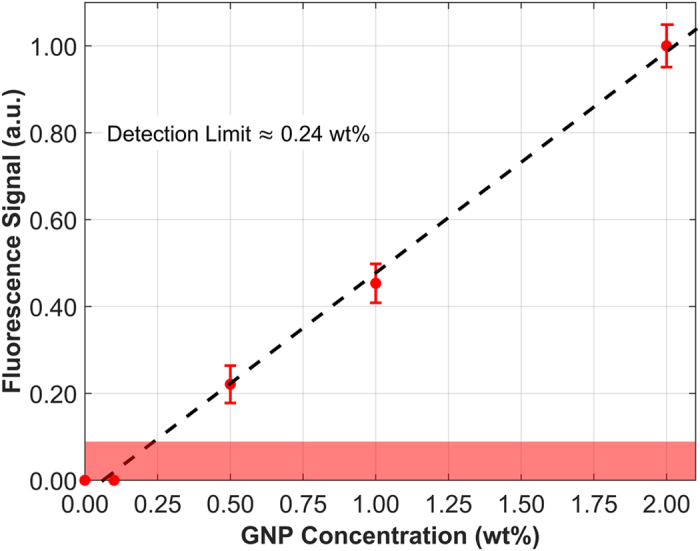
Calibration curve depicting the net x-ray fluorescence signal (a.u., arbitrary units) as a function of GNP concentration. The shaded area represents signal less than 1.96 times the standard deviation of the background (noise level), which was therefore considered to be noise rather than true signal. The intersection of the shaded region and the linear fit represents the detection limit (~0.24 wt%).

**Figure 4:**
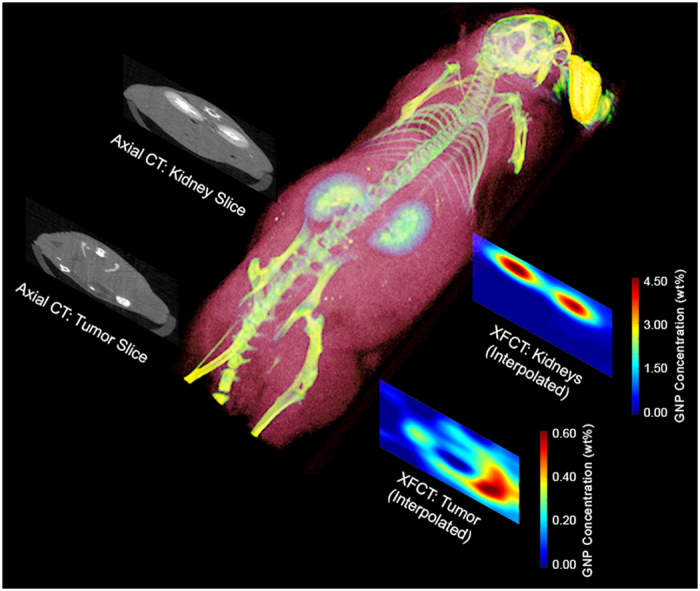
3D volume rendering of the mouse from postmortem CT data along with smoothed XFCT images (bicubic interpolation) and corresponding axial CT images of the kidney and tumor slices. This figure illustrates the possibility of quantitative multimodal imaging of GNP distributions in an animal using XFCT and conventional CT together.

**Figure 5:**
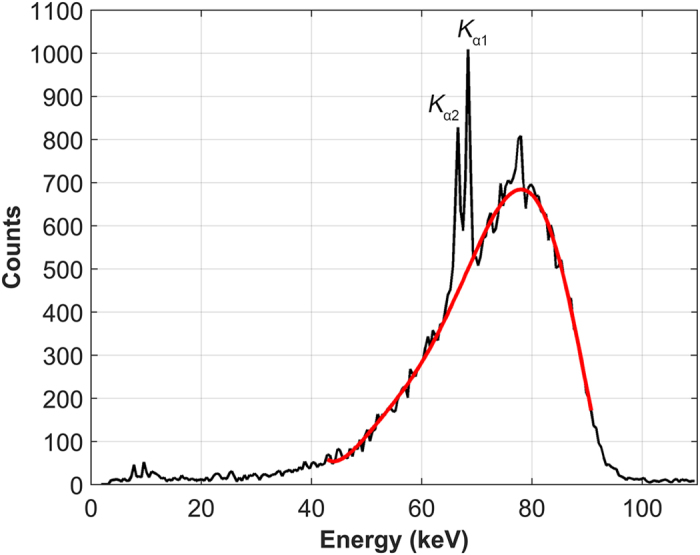
Illustration of data processing procedure to extract XRF signal from scatter/XRF spectrum acquired by detector. This particular spectrum was from a single 15–s acquisition of a GNP-containing region in the kidney of the mouse. Each spectrum was corrected for detection efficiency. A polynomial fit was applied to the Compton scatter background to facilitate extraction of signal from gold *K*_α_ XRF peaks (68.8 and 67.0 keV).

A calibration curve (Fig. 3) was obtained under the same imaging geometry as that used for XFCT by measuring the XRF signal for various known concentrations of GNPs ranging from 0.01 to 2 wt% suspended in PBS. For these measurements, containers filled with the GNP-containing solutions were embedded within a 3-cm-diameter plastic phantom acting as a surrogate for a small animal; more information about this phantom can be found in our previous experimental work^24^. The data were processed according to the procedure described in the previous section, including use of the Compton-scatter-based attenuation correction algorithm. A linear fit of the data was used to correlate net XRF signals with GNP concentration. In addition, the material detection limit (system sensitivity) was determined by defining the minimum signal needed to obtain a net XRF signal above the 95% confidence interval; that is, only peak signal at least 1.96 times the standard deviation of the background was considered to be true signal. The detection limit was found to be a GNP concentration of approximately 0.24 wt%, consistent with previously obtained computational predictions^26^.

### ICP-MS analysis of explanted tissues for quantification of GNPs

Immediately after image acquisition, organs were extracted, and the levels of elemental gold in tissues were analyzed using ICP-MS (Varian 810 Quadrupole ICP-MS system; Varian, Palo Alto, CA), with a detection limit of <0.4 ng/L (<0.0004 ppb) for gold and precision of <3% according to the manufacturer’s specifications. In brief, the collected tissue samples were frozen, lyophilized, and digested in 1 mL of aqua regia (250 μL of HNO_3_, 750 μL of 37% HCl) for 72 h to dissolve the GNPs. The samples were then diluted to 10 mL with 2% HCl and centrifuged, and clear supernatant was collected and filtered before elemental analysis using ICP-MS. A series of calibration standards (5, 10, 20, 50, 100, and 200 ppb) were prepared from a single-element standard (Inorganic Ventures, Christiansburg, VA) to construct a calibration line for offline data reduction. Sensitivity drift was monitored and corrected through the analysis of drift in the control solutions of known concentrations of the elemental standard.

## Additional Information

**How to cite this article**: Manohar, N. *et al.* Quantitative imaging of gold nanoparticle distribution in a tumor-bearing mouse using benchtop x-ray fluorescence computed tomography. *Sci. Rep.* **6**, 22079; doi: 10.1038/srep22079 (2016).

## Figures and Tables

**Table 1:** CT and XFCT CNRs for regions of interest within a single axial slice covering the kidneys and another slice covering the tumor region.

Organ/Region of Interest	CNR	
	CT	XFCT
Left kidney	1.83	174
Right kidney	1.80	189
Tumor	0.0295	15.6
